# Dietary Restriction for Kidney Protection: Decline in Nephroprotective Mechanisms During Aging

**DOI:** 10.3389/fphys.2021.699490

**Published:** 2021-07-06

**Authors:** Nadezda V. Andrianova, Marina I. Buyan, Anastasia K. Bolikhova, Dmitry B. Zorov, Egor Y. Plotnikov

**Affiliations:** ^1^A.N. Belozersky Institute of Physico-Chemical Biology, Lomonosov Moscow State University, Moscow, Russia; ^2^Faculty of Bioengineering and Bioinformatics, Lomonosov Moscow State University, Moscow, Russia; ^3^V.I. Kulakov National Medical Research Center of Obstetrics, Gynecology and Perinatology, Moscow, Russia

**Keywords:** caloric restriction, kidney injury, ischemia/reperfusion, nephrotoxicity, therapy, AMPK, sirtuins

## Abstract

Dietary restriction (DR) is believed to be one of the most promising approaches to extend life span of different animal species and to delay deleterious age-related physiological alterations and diseases. Among others, DR was shown to ameliorate acute kidney injury (AKI) and chronic kidney disease (CKD). However, to date, a comprehensive analysis of the mechanisms of the protective effect of DR specifically in kidney pathologies has not been carried out. The protective properties of DR are mediated by a range of signaling pathways associated with adaptation to reduced nutrient intake. The adaptation is accompanied by a number of metabolic changes, such as autophagy activation, metabolic shifts toward lipid utilization and ketone bodies production, improvement of mitochondria functioning, and decreased oxidative stress. However, some studies indicated that with age, the gain of DR-mediated positive remodeling gradually decreases. This may be an obstacle if we seek to translate the DR approach into a clinic for the treatment of kidney diseases as most patients with AKI and CKD are elderly. It is well known that aging is accompanied by impairments in a huge variety of organs and systems, such as hormonal regulation, stress sensing, autophagy and proteasomal activity, gene expression, and epigenome profile, increased damage to macromolecules and organelles including mitochondria. All these age-associated changes might be the reasons for the reduced protective potential of the DR during aging. We summarized the available mechanisms of DR-mediated nephroprotection and described ways to improve the effectiveness of this approach for an aged kidney.

## Dietary Restriction: Introduction

Caloric or dietary restriction (DR), which is defined as reducing nutrient intake without malnutrition, is considered one of the most proven approaches to extend life span and to delay deleterious age-related physiological changes and age-related diseases in animals (Speakman and Mitchell, 2011). Positive effects of DR were shown for myocardial infarction (Rohrbach et al., 2014), degenerative brain diseases (Fusco and Pani, 2013), hypertension (Han and Ren, 2010), diabetes mellitus (Sathananthan et al., 2015), cancer (Alidadi et al., 2020), and others. The influence of various dietary protocols on physiological effects is extensively studying in patients with acute kidney injury (AKI) and chronic kidney disease (CKD; Lambert et al., 2020; Müller et al., 2020), as well as in experimental models (Singh and Krishan, 2019).

The effectiveness of DR in the prevention of aging and age-related diseases can be explained by the effect of hormesis (Kouda and Iki, 2010; Shushimita et al., 2016), since it is known that the induction of moderate stress activates the adaptive responses of cells and organs, reducing the intensity of damage when the organism is exposed to more severe stress. To date, DR has been shown to trigger a large number of signaling cascades that lead to changes in cellular metabolism to adapt to low-intake conditions. The same goes for gene expression in all tissues (Ma et al., 2020), as well as the epigenetic profile (Gensous et al., 2019). Along with many other mechanisms, adaptation to reduced nutrient intake is achieved through IGF-1R and mTOR complexes signaling (Johnson, 2018). Inhibition of the IGF-1 receptor during DR leads, in particular, to inhibition of cell proliferation, activation of autophagy but simultaneously increasing in antioxidant systems activity. Another involved metabolic regulator is AMPK, which is a fast-responding sensor of nutrients in cells, activated when AMP/ATP ratio is increased (Cantó and Auwerx, 2011). AMPK phosphorylates FoxO transcription factor, which leads to the activation of genes of stress response and the use of energy sources other than glucose (Greer et al., 2009). Another important nutrient sensor is NAD^+^-dependent deacetylases sirtuins, which deacetylated a large number of targets, including transcription factors, thereby regulating its activity (Guarente, 2013).

Based on molecular mechanisms, DR and its mimetics have been supposed as one of the most promising approaches to the treatment of various pathologies, especially those related to old age. The attention of this review is focused on describing the effects of DR on renal injury in young and old animals, and comparison of the effectiveness of DR in animals of different age. Since limiting the food intake without changing the ratio of nutrients content is the most common way of DR, we focused our attention on this type of restriction. Of note, the timing, duration, and composition of vitamins and minerals in food during DR can significantly influence the effects of this approach so that these parameters should be carefully controlled.

## Dietary Restriction as a Nephroprotective Approach in Young Animals

Although the protective mechanisms of DR were discovered as early as in 1935 (McCay et al., 1935) and have been intensively studied, there are quite a few studies showing the effects of DR on the kidneys in normal or pathological conditions. While ischemic injuries predominate among the causes of AKI (Mehta et al., 2004), the protective effect of short-term DR on the kidneys exposed to ischemia/reperfusion (I/R) was revealed only in 2010 (Mitchell et al., 2010).

In this study, young mice underwent 30% DR for 4 weeks prior to I/R that caused an attenuation of the severity of AKI and reduced postoperative mortality. In the kidneys of DR-treated mice, alleviation of I/R-induced acute tubular necrosis and release of lactate dehydrogenase were observed, which was associated with a change in the transcriptional profile (Mitchell et al., 2010). In addition, this work pointed to the importance of continuous DR, since the return of *ad libitum* feed before I/R significantly reduced the nephroprotective properties of DR. Subsequently, the protective effects of DR in ischemic AKI have been shown in a number of studies. We compared different DR protocols (25 and 35% DR for 4 weeks and 100% DR for 3 days) and found that 35% DR provided the maximum protective effect against I/R injury (Andrianova et al., 2020). Later, it was shown that reduced food intake for 6 weeks regardless of time or fat affords protection of young mice against renal I/R (Reynolds et al., 2019).

In young animals, very short periods of DR also showed protective properties in various models of AKI. For example, fasting for 3 days before I/R injury preserved rats from damage to the tubules and renal functional decline by increasing antioxidant defense and maintaining mitochondrial structure and functions (Rojas-Morales et al., 2019). Short-term preoperative 30% DR and 3-day fasting protected against renal I/R during kidney transplantation, both reducing mortality and improving the transcriptional profile (Jongbloed et al., 2017). The protection was also found in a one-week DR in a rat model of kidney I/R injury, where DR improved renal function, suppressed tubular injury, prevented activation of ERK1/2, and inhibited the development of interstitial fibrosis, as well as reduced blood glucose, increased β-hydroxybutyrate, improved antioxidant protection, and DRP1-mediated mitochondrial fragmentation (Rojas-Morales et al., 2020).

Studies in young rats have shown that DR has a protective effect not only against ischemic renal injury but also in models of drug-induced AKI (Perazella, 2019). Thus, DR ameliorated acute cisplatin- and cadmium nephrotoxicity (Shaikh et al., 1999; Estrela et al., 2017). DR with different protein and fat content for 3 days before or after cisplatin-induced AKI reversed the nephrotoxic effect of cisplatin treatment and was associated with phosphorylation of survival kinases PI3K/Akt and ERK-1/2, decreased level of stress kinase JNK, and improved physiological outcomes (Gunebakan et al., 2020). The protective properties of DR against cisplatin-induced AKI were also confirmed using multi-layered omics data (transcriptome, proteome, and N-degradome) correlated with functional parameters (Späth et al., 2019). Such bioinformatic analysis revealed mRNA-independent changes in proteome that affect the extracellular matrix, mitochondria, and membrane transporters associated with the protective properties of DR. The positive effects of DR are manifested not only in AKI but also in models of CKD (Gumprecht et al., 1993) and diabetic nephropathy (Kume and Koya, 2015). Thus, DR protects the kidney tissue not only from acute injuries but also from chronic ones.

To date, only some molecular mechanisms of the beneficial DR effects in renal tissue have been proposed. Using novel methods of comparative analysis of microarray data, detailed comparisons of DR-mediated changes in various tissues were carried out resulting in the identification of the 28 most affected genes. These genes characterized common responses to DR and involved both activation and inhibition of stress-response pathways (Swindell, 2008). DR was shown to ameliorate kidney I/R injury through the PGC-1α-eNOS pathway, activation of SIRT1 and AMPK, and enhanced autophagy (Lempiäinen et al., 2013). Our study also revealed that the protective properties of DR are associated with activation of the autophagosomal-lysosomal system, normalization of mitochondrial functioning, and decrease of oxidative stress (Andrianova et al., 2020). Kidney protection by DR may also be mediated by a decrease in the level of mannan-binding lectin, which initiates the lectin pathway of complement system activation (Shushimita et al., 2015).

While the beneficial effects of DR are partially mediated by above-mentioned molecular changes, there are many other protective mechanisms related to DR. For instance, reduced protein intake during DR significantly improves the prognosis of AKI due to normalizing intraglomerular pressure and glomerular hyperfiltration (Ko et al., 2017). One more important factor that could interact with DR effects is the microbiome and its metabolites. Some studies postulate that gut microbiota may be affected by DR and can mediate DR effects on metabolism and hormone regulation (Wang et al., 2018). Moreover, fecal transplantation from mice with DR significantly reduced body weight and obesity in recipient thereby DR positively changes microbiome composition (Pérez-Matute et al., 2020).

Eventually, DR has shown impressive effectiveness in the treatment of experimental kidney pathologies, which makes it possible to translate this approach into clinical practice, especially in conditions when the risk of AKI is increased, for example, during cardiac surgery (Grundmann et al., 2018). In addition, DR improves transplant outcomes from young donors by reducing ischemic damage during transplantation (van Ginhoven et al., 2011; Jongbloed et al., 2020). To improve kidney function, a low-calorie or ketogenic diet is recommended for people with obesity accompanied by mild renal insufficiency (Bruci et al., 2020). Dietary interventions are also recommended for patients with CKD and can ameliorate glomerular filtration rate, lower blood pressure, and serum cholesterol levels, thereby improving health-related quality of life (Palmer et al., 2017).

## Dietary Restriction in Old Animals

While the potential of DR to reduce the severity of AKI has been confirmed in experiments with young animals, the largest and most vulnerable group of patients with AKI and CKD is represented by the elderly. The average age of patients with AKI tends to 65 years and steadily decreases every year due to an increase in life expectancy (Mehta et al., 2015). Accordingly, experimental models should use old animals to consider the structural, functional, and molecular changes observed in kidneys during aging. Aging not only affects the morphology and metabolism of kidney tissue but also worsens ischemic tolerance and increases tissue vulnerability to injury (Rosner et al., 2018), so experiments in old animals are more preferable for the development of therapy for AKI and CKD.

On the other hand, it has been suggested that life-long DR is most effective in preventing the development of age-related changes in the renal tissue meaning dieting should begin at a young age to protect the old kidney. Indeed, DR started at an early age substantially improved age-associated renal histological abnormalities and survival (Bras and Ross, 1964). Life-long DR prevented the development of gradually increasing morphological changes in the kidney, such as glomerular lesions, thickening of the basement membrane, and tubular dilatation. Moreover, long-term DR has been proved to significantly extend life span and to ameliorate age-related polycystic kidney disease and CKD (Tomobe et al., 1994; Warner et al., 2016; Yoshida et al., 2018). In rats, DR for 30 months with reduced total calorie intake or protein content retarded the severity of age-related chronic nephropathy (Masoro et al., 1989) and reversed the aging-related loss of protein in urine (Teillet et al., 2000).

However, some studies have demonstrated that not only life-long DR but also short-term DR protocols are effective against age-associated abnormalities in kidneys. DR initiated in rats of 6 months of age was as effective as food restriction initiated at 6 weeks of age in slowing the progression of chronic nephropathy (Maeda et al., 1985). Moreover, DR initiated in middle-aged rats, before the onset of significant age-related changes, as well as long-term DR effectively reduced glomerulosclerosis and tubular atrophy, prevented the formation of interstitial fibrosis, thickening of the vascular wall, and the decrease of cytochrome c oxidase expression (McKiernan et al., 2007; Podkowka-Sieczka et al., 2009). The manifestation of the positive effects of DR even at the debut in adulthood encourages the study of short-term DR protocols due to the great interest in clinical practice which is faced with the complexity of life-long dieting in people.

To date, an active investigation of the effects of DR on old animals continues. Transcriptome analysis of renal tissue after life-long DR showed that DR modulates the expression of many genes in old rats and has benefits for kidney function (Chen et al., 2007). It was found that the expression of 92 genes changed during aging and was reversed by DR for 22 months, including claudin-7, Kim-1, and MMP-7 (Chen et al., 2007). In addition, the study of the single-cell transcriptional landscape after 9 months of DR revealed significant changes in the expression profile of a large number of genes, including those affecting the process of cellular senescence, stem cell depletion, chronic inflammation, and cell-to-cell communication (Ma et al., 2020). Adult-onset DR for 6 months also dramatically changed the gene profile, significantly reduced urinary 8-isoprostane and protein carbonyl in the kidney and downregulated inflammatory response pathway (Chen et al., 2008).

The molecular mechanisms of DR in old organisms look similar to young animals. For instance, DR in both young and old rats reduced the pro-inflammatory response from NF-kB and AP-1 and normalized the network of these transcription factors in the renal tissue of old animals (Jung et al., 2009). Old rats that underwent long-term DR showed higher expression of sirtuin 1 in kidney tissue, a higher degree of autophagy activation, shifted acetylation status of transcriptional growth factors to a more deacetylated state, and an improvement in the functional activity of mitochondria (Kume et al., 2010). Rats exposed to DR in adulthood had lower levels of renal fibrosis and levels of extracellular matrix proteins type IV collagen and fibronectin (Jiang et al., 2005) that is believed to be achieved by depletion of miR-21 expression (Liu et al., 2020). Similarly, DR for 8 weeks reduced renal expression of α-smooth muscle actin, lowered p16, p21, and SA-β-gal levels and activated AMPK/mTOR signaling pathway (Ning et al., 2013b; Dong et al., 2017).

Unsurprisingly, DR affected energy metabolism and mitochondrial functions. The beneficial effects of adult-onset DR manifested in a decreased accumulation of abnormally folded proteins in the kidney mitochondria in old animals (McKiernan et al., 2007). DR for 3 months in old rats significantly upregulated arginase II activity, which normally regulates urea cycle, polyamine, proline, glutamate synthesis, and production of nitric oxide (Majaw and Sharma, 2017). Late-onset DR reversed the age-related decline of malate–aspartate shuttle enzymes in the kidneys (Goyary and Sharma, 2008). The spectrum of mitochondria-associated effects of DR also included as follows: increase in the levels of the anti-apoptotic protein Bcl-X_L_, normalization of mitochondrial ultrastructure, diminished oxidative stress (Cui et al., 2013; Andrianova et al., 2020), abrogation of age-associated expression of a pro-apoptotic Bax protein, caspase-3 activation, and activation of PARP polymerase (Lee et al., 2004).

Note that despite a number of positive changes at the molecular level caused by DR, there are only a few studies that describe the effects of DR on kidney function in AKI. Thus, old rats that received 60% of normal food intake for 2 months showed decreased blood urea nitrogen and serum creatinine levels, reduced renal tubular necrosis, and lower incidence of activated caspase-3 and TUNEL-positive cells in kidneys after cisplatin-induced nephrotoxicity (Ning et al., 2013a). However, we showed in the renal model of I/R, that in old rats DR for 1 or 2 months was not as effective as in young and did not reduce AKI measured by the level of serum creatinine and urea, as well as NGAL level in urine (Andrianova et al., 2018, 2020). The loss of DR effectiveness during aging is more likely to have a gradual pattern since in 12-month-old rats DR still demonstrated some nephroprotective effect, but to a lesser extent than in young animals (Andrianova et al., 2020).

## Possible Mechanisms of Dietary Restriction Impairment During Aging

Most fruitful experimental studies of various DR protocols have been conducted in young animals (Singh and Krishan, 2019), while a few studies of DR protection in renal injury have shown a decline in beneficial effects with age (Andrianova et al., 2018, 2020). This raises the question of implementation of DR in clinical practice since elderly people predominate among patients with AKI, so additional studies are needed to improve the effectiveness of DR for the aged kidney.

The loss of protective effects of therapeutic methods with age is a significant problem not only for DR but also for other treatment approaches. Earlier, a similar loss of positive impact was described when applying ischemic pre- and postconditioning for kidney and heart of old animals (Abete et al., 2002; Boengler et al., 2008; Chen et al., 2014; Jankauskas et al., 2017). Similarly, in elderly mice, DR did not improved impaired wound healing (Reed et al., 1996). We hypothesize that the loss of protective properties is a natural and common phenomenon for the majority of therapeutic approaches (Jankauskas et al., 2018).

Thus, not all tissues and systems show improvements during DR with age. For instance, despite the observation that DR ameliorated the state of blood vessels and their response to the vasoconstrictive effect of endothelin-1 in young rats, such positive effects were absent in old rats after short-term DR (Amor et al., 2017). Moreover, moderate DR stimulated angiogenesis to a very small extent in 24-month-old rats (Facchetti et al., 2007), whereas it is angiogenesis during DR that is supposed to prevent vascular impairment in the heart and brain in younger animals (Csiszar et al., 2013). The state of the immune system in old animals did not improve during DR either. It was found that long-term 30% DR did not reduce DNA damage in lymphocytes (Gedik et al., 2005), and old mice maintained on life-long 40% DR were even more prone to influenza infection and had worse survival compared to old *ad libitum* mice (Gardner, 2005).

Insufficient efficiency of DR in old animals is also observed for the hormone levels. In contrast to the leptin level, which decreased during DR in both young and old animals, the adiponectin concentration increased only in young rats (Rohrbach et al., 2007), so DR was unable to fully improve the functioning of adipocytes while aging. Moreover, short-term DR had different effects on lipogenic enzymes in the white adipose tissue of young and old rats demonstrating a reduced adaptation of old animals to a restricted diet (Wronska et al., 2014). Only life-long DR increased the content of thyroid hormones of rhesus monkeys, whereas short-term DR did not affect the level of thyroid hormones in old animals (Roth et al., 2002).

The observed loss of protective properties of therapies can be partially explained by various age-dependent changes that accumulate in all tissues and affect the functioning and tolerance of organs (Rezzani et al., 2012). Thus, in the kidney, both structural changes consisting of a decrease in the number of functioning nephrons, degenerative changes in the proximal tubules, glomerulosclerosis, and changes in molecular pathways are observed, e.g., increased expression of renal pathologies-associated genes, claudin-7, KIM-1, and metalloproteinase (Chen et al., 2007). Aging also leads to significant epigenetic changes at all levels of chromatin and DNA organization (López-Otín et al., 2013; Kane and Sinclair, 2019).

Significant metabolic shifts accompany aging leading to impaired lipid and carbohydrate metabolism and loss of nutrient-sensing pathways (Ehrhardt et al., 2019). There are malfunctions in DR-mediated pathways in the elderly including those with IGF-1R, AMPK, sirtuins, and mTOR (Bettedi and Foukas, 2017). Aging is also associated with cellular senescence, which causes such detrimental phenomena as chronic inflammation (Furman et al., 2019), impaired tissue remodeling after injury, and contributes to a decline of regenerative potential (Di Micco et al., 2021). The accumulation of senescent cells could lead to greater sensitivity to injury and reduced tissue repair.

Endocrine functions are also disrupted with age and the kidneys are no exception (Bolignano et al., 2014). In particular, the content of the components of the renin-angiotensin system and the levels of aldosterone decrease in blood plasma (Yoon and Choi, 2014). Although the concentration of erythropoietin in the blood is higher in the elderly compared to the young, there is no pronounced erythropoiesis in response to a drop in hemoglobin levels in elderly (Ferrucci et al., 2007; Garimella et al., 2016). The transformation of vitamin D into the active form, which largely occurs in the kidneys, also suffers with aging (Armbrecht et al., 1980). Despite an increase in the levels of adiponectin in the blood of old organisms, adiponectin-dependent regulation is disrupted and aging is paradoxically associated with the loss of the functionally active isoform of the hormone (Gulcelik et al., 2013).

An important age-related disorder is the deterioration of cellular quality control systems for proteins and organelles. With age, both the dysfunction of the autophagic-lysosomal system (Mizushima et al., 2008) and the proteasome machinery have been described (Sun-Wang et al., 2020). This inevitably leads to the accumulation of aggregates of misfolded proteins (Hipp et al., 2019) and dysfunctional organelles, particularly mitochondria (Payne and Chinnery, 2015). The accumulation of poorly functioning mitochondria is dangerous for cells as it can cause increased oxidative stress (Liguori et al., 2018). Changes in the morphology of mitochondria with age have been described for many organisms (Sastre et al., 1996), as well as the accumulation of age-associated mitochondrial proteins (Cui et al., 2012) and a decrease in the transmembrane potential (Serviddio et al., 2007).

## Conclusion

**Figure 1:**
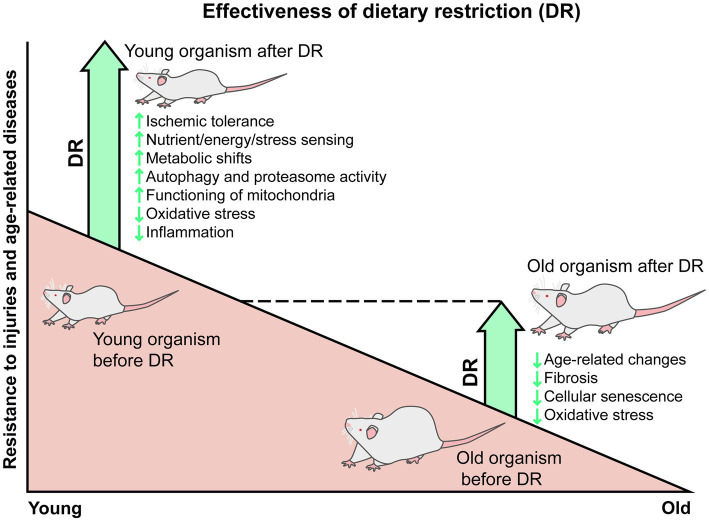
Gradual reduction of the DR protective effects from injuries and age-related changes. In young animals, DR is accompanied by many positive changes, such as enhanced response of nutritional and stress-sensing pathways, metabolic shifts toward lipid utilization and ketone bodies production, autophagy and proteasome activation, improvement in mitochondria functioning, reduced oxidative stress and inflammation, and subsequent resistance to injuries, including ischemic ones. In old animals, DR is associated with a smaller range of positive changes, such as delaying age-related changes and the formation of fibrosis, reducing the number of senescent cells, oxidative stress, and chronic inflammation. The adult-onset DR does not improve all age-related impairments in all organism’s systems, so old animals kept on DR do not fully reach the state of young healthy organisms.

Thus, DR is considered a promising approach for the treatment of various age-related diseases, including AKI and CKD. However, some studies reveal a gradual loss of effectiveness of DR with age, which is alarming given the advanced age of patients with AKI and CKD. The adult-onset DR leads to a number of positive changes, but they affect much fewer pathways, so old organisms after DR cannot develop such high improvements and tolerance as young healthy organisms do (Figure 1). We postulate that the loss of the nephroprotective properties of DR is part of a natural and general phenomenon inherent in most therapeutic approaches, and this may be due to the accumulation of deleterious changes at the physiological, cellular, and molecular levels during aging. These changes lead to the deterioration of a stress response and reduced adaptation to limited calorie intake. Thereby, the further studies of the DR mechanisms are required to improve the DR effectiveness in elderly organisms.

## Author Contributions

NA and EP conceived the manuscript and developed the idea. NA wrote the review. MB and AB helped to collect the data and drafted the manuscript. EP and DZ critically revised and improved the content of the manuscript. All authors contributed to the article and approved the submitted version.

### Conflict of Interest

The authors declare that the research was conducted in the absence of any commercial or financial relationships that could be construed as a potential conflict of interest.
